# Enteric Fever in the Army

**Published:** 1905-11-18

**Authors:** 


### The Hospital.

A Journal of

The Medical Sciences and Hospital Administration.

Vol. XXXIX.—No. 999. SATURDAY, NOVEMBER 18, 1905.

### Enteric Fever in the Army.

The indefatigable Dr. Leigh Canney has once more brought the subject of the prevention of enteric fever in the Army, and specially in an army engaged in active military operations, prominently before the notice of the public; and on this occasion he has been fortunate, not only in his audience at the Royal United Service Institution, but also in an adversary by whom, as a representative of the official art "how not to do it" his teachings have lately been assailed. A fortnight or so before the date of Dr. Canney's lecture at the institution, Surgeon-General Sir Thomas Gallwey, Principal Medical Officer of the Forces in India, attacked him in a letter which covered nearly two columns of the *Times*; and which, had it not been for the signature, we should have attributed rather to a "combatant" officer of the old school than to a member of the medical profession. Sir Thomas set himself to argue against the well-established fact that excrementally polluted water is always dangerous, because such pollution is always liable to involve the special pollution with the excretions of fever patients; and he dealt with the question as if he supposed "filth," independently of one special form of filth, to be the supposed cause of the diffusion of the disease. Everyone admits, we presume, that there are other channels besides polluted water through which enteric fever may be diffused; but all experience points to the conclusion that, especially in the case of armies in the field, polluted water is of more importance than all other channels put together. Dr. Leigh Canney has at least the merit of having suggested a perfectly clear and definite line of policy by which all water drank by troops on service would be sterilised and rendered safe. He has shown that a single mule would supply the transport required for this purpose by a unit of 100 men, and that nine minutes would allow the sterilised water to become cool enough to drink. But he has rightly insisted upon the fact that no exceptions to the use of the sterilised water must be allowed if security is to be obtained, that for this purpose the attachment to each unit of a small staff of trained non-combatant men is essential, and that it must be made a matter of military duty for every man to co-operate in the preservation of himself and his comrades from the disease which has hitherto been the chief scourge of armies. In order that such co-operation may become effective, it is necessary that both officers and men should be better instructed in regard to sanitation than they are at present; or, in other words, that they should be made to understand the nature of the risks against which they are called upon to exert some common sense for their own protection. They are taught to take cover from the fire of the enemy; and they must be taught to take cover from the attacks of an enemy far more insidious and more dangerous. Under the time-honoured arrangements now in force in the British service, ten men are disabled by enteric fever for every one that is disabled by shrapnel or rifle shot.

A common argument against Dr. Leigh Canney's proposals, when they are discussed by officers, whether combatant or medical, of "the old school," is to exaggerate the urgency and intensity of the demand for fluid. They talk of "raging thirst," and that sort of nonsense; just as if the real demands of the human system for liquid were not quite measurable, and; within certain limits, quite controllable. In the Crimea, Sir Colin Campbell, as he then was, stopped the Highland Brigade by one word when the men were rushing to drink at a stream close by, and made them halt, dress, and go quietly and in order. What he did could be done also by others; and commanding officers of intelligence even now do a good deal to check the mere bad habit of excessive drinking into which soldiers are very liable to fall. There is a sort of natural association between the control of appetites and the capacity for arduous and strenuous exertion; insomuch that the men who are capable of the latter, as all soldiers should strive to be, are the most accustomed to exercise the former also. Whatever may be thought of Dr. Leigh Canney's proposals, or whatever difficulties may stand in the way of adopting them, it is at least certain that nothing but good could arise from teaching soldiers the precise nature of the chief danger attendant upon military service. It is probable that, as a rule, they do not in the least realise the facts of the case or the nature of the risks which they incur. They are no doubt rendered sceptical by the circumstance that they often drink thick or muddy water with impunity, and hence obtain the impression that any chances of infection arising from it have been exaggerated by the doctors and need not be regarded. They do not realise that the latrine system of a camp exposes all water to one particular kind of pollution; and that, through this kind of pollution, the first case of enteric fever may poison a whole regiment. Soldiers, as a rule, are rough and ignorant men, and it can be no matter for surprise that they show no more enlightenment than the municipality of a cathedral city. The municipality of Lincoln have known for years that the water supply under their control was exposed to excremental pollution, and they have repeatedly been warned that, sooner or later, this would entail the presence of the enteric fever bacillus. When the bacillus came, and the fever with it, they spoke as if they were the victims of undeserved and unforseen misfortune. The "military" authorities have for some time treated the warnings and suggestions of Dr. Canney very much upon the same principle.

